# Expressions of vascular endothelial growth factor A, mucin-1, colony-stimulating factor-1, heparin-binding epidermal growth factor-like growth factor, and fibroblast growth factor 2 genes in the female reproductive tracts of women with ectopic pregnancy: A case-control study

**DOI:** 10.18502/ijrm.v21i10.14535

**Published:** 2023-11-24

**Authors:** Sima Golkar, Zahra Chekini, Fatemehsadat Amjadi, Parvaneh Afsharian, Aida Najafian, Firouzeh Ghaffari, Reza Aflatoonian

**Affiliations:** ^1^Department of Genetics Science, Faculty of Basic Sciences and Advanced Technologies in Biology, University of Science and Culture, ACECR, Tehran, Iran.; ^2^Department of Endocrinology and Female Infertility, Reproductive Biomedicine Center, Royan Institute for Reproductive Biomedicine, ACECR, Tehran, Iran.; ^3^Department of Anatomy, School of Medicine, Iran University of Medical Science, Tehran, Iran.; ^4^Department of Genetics, Reproductive Biomedicine Research Center, Royan Institute for Reproductive Biomedicine, ACECR, Tehran, Iran.; ^5^Department of Infertility, Shariati Hospital, Tehran University of Medical Sciences, Tehran, Iran.

**Keywords:** Ectopic pregnancy, Gene expression, Vascular endothelial growth factor A, Mucin-1, Colony-stimulating factor-1, Heparin-binding epidermal growth factor-like growth factor, Fibroblast growth factor 2.

## Abstract

**Background:**

Ectopic pregnancy (EP) is defined as embryo implantation in a location other than the uterine cavity.

**Objective:**

We aimed to evaluate the expression of several genes, which may play a role in EP, in the ampulla region of fallopian tubes and endometrial tissue of women with EP.

**Materials and Methods:**

In this case-control study, 5 women who underwent salpingectomy due to EP, comprised the 5 pseudo-pregnant women as a control group. These participants referred to the Royan Institute, Shariati, and Arash hospital, Tehran, Iran during 2019-2021. We evaluated the expressions of vascular endothelial growth factor A*, *mucin-1*, *colony-stimulating factor-1*,* heparin-binding epidermal growth factor-like growth factor* (HBEGF),* and fibroblast growth factor 2 genes in the fallopian tube and endometrium of EP cases by real-time polymerase chain reaction using specific primers.

**Results:**

The vascular endothelial growth factor expression was significantly higher in the ampulla region of the controls. However, no significant differences were observed in endometrial tissue. Assessments of colony-stimulating factor-1 and fibroblast growth factor 2 showed no significant differences between the case and control groups. *HBEGF* showed significantly higher expression in the ampulla region of EP cases, but no significant difference was observed in *HBEGF* expression in the endometrial tissues of the study groups. Mucin-1expression was significantly higher in both study regions of the EP cases.

**Conclusion:**

Our results have strongly suggested that these genes play important roles in proper implantation, and disruptions in their expression patterns could lead to EP. However, more studies are needed to confirm the current findings.

## 1. Introduction

Ectopic pregnancy (EP) is the implantation of an embryo somewhere other than the uterine cavity. More than 98% of EPs occur in the fallopian tube (1, 2). EP has become the main cause of maternal morbidity and mortality in the first trimester of pregnancy (3, 4). There is no clear understanding of the molecular mechanisms responsible for EP (1). However, several researchers have suggested the embryo's arrest within the fallopian tube due to impaired tubal transport as a result of tubal microenvironment alterations leading to inappropriate implantation (1, 5). Several genes have been identified that could affect the tubal environment as well as play a role in endometrial receptivity. These genes include vascular endothelial growth factor A (*VEGF-A)*, Mucin-1 (*MUC1*), colony-stimulating factor-1 (*CSF1*), heparin-binding epidermal growth factor-like growth factor (*HBEGF*), and fibroblast growth factor 2 (*FGF2*) (6-10).

Angiogenesis is one of the most important aspects of successful implantation (11). The results of previous studies have shown that *VEGF-A,* as a potent angiogenic factor, has a significantly lower expression in the fallopian tubes of the EP cases (2, 12). *MUC1* acts as an epithelial apical surface glycoprotein, which should be removed for successful interaction between blastocyst and endometrium during implantation (13, 14). High levels of *MUC1* inhibit cell-cell adhesion, controversy its lower expression leading to EP compared to pseudopregnant women (6, 13, 14). *CSF1* is a regulator of macrophage growth and differentiation that plays a significant role in reproduction and pregnancy (15, 16).

HBEGF is a molecular mediator which signals between the endometrium and trophoblast cells. The study results have demonstrated that *HBEGF* might play role in the attachment and penetration steps of embryo implantation and could be a helpful marker for the implantation window (10). *FGF2* is one member of the FGF family, which are multifunctional regulators of different cellular processes including differentiation, migration, and growth (9). Previous investigations have shown that endometrial expression of FGF2 did not change between days 1 and 12 of pregnancy; however, the expression was higher in the proliferative phase rather than the secretory phase (8).

Here, we evaluated the expression of the *VEGF-A, MUC1, CSF1, HBEGF,* and* FGF2* genes in the fallopian tubes and endometria of women with EP in order to investigate the role of genes in the decreasing the endometrial receptivity and increasing the ectopic embryo implantation in the fallopian tube.

## 2. Materials and Methods

### Subjects

In this case-control study, 5 women who underwent salpingectomy due to EP comprised the case group. These participants referred to the Royan Institute, and Shariati, and Arash hospital, Tehran, Iran during July 2019-August 2021. The ampulla region of the fallopian tube and endometrium were obtained from each case for further investigation. For the control group, as our team previously described, we obtained tissues from 5 cases that underwent hysterectomies due to benign gynecological situations without affecting the tubes. The control group were fertile women with regular menstrual cycles, and no evidence of any pathological tubal disorders. To induce normal pregnancy hormonal conditions, we created a state of pseudo-pregnancy according to the previous study (2). This procedure is harmless for these women, as previously stated by other studies (17, 18). All EP cases included the following criteria, age 20-37 yr, regular menstruation, and normal body mass index. The exclusion criteria were as follows, receiving hormonal drugs for 3 months before surgery, salpingitis, smoking, diagnosis of heterotopic pregnancy with vaginal ultrasound, receiving methotrexate, history of using intrauterine devices, history of previous EP, uterine diseases such as (endometriosis, polycystic ovarian syndrome, uterine anomalies, myoma), history of abdominal surgery, and thyroid disease. We removed at least 1 cm of tissue from the site where the embryo was implanted to prevent the collection of any embryonic or trophoblastic tissues.

### RNA extraction using TRIzol and reverse transcription polymerase chain reaction (RT-PCR)

Total RNA extraction was performed using TRIzol (TRI Reagent, Sigma, Pool, UK) based on the instructions provided in our previous studies (2, 19). After total RNA extraction, we quantified the RNA concentrations by spectrophotometric analysis to assess the quality of the extracted RNA.

The process of creating the first strand of cDNA involved using oligo (dT) primers and the superscript II reverse transcriptase system (Fermentas, Sankt Leon-rot, Germany). To synthesize the cDNA, a master mix was prepared containing 1 µg RNA and 1 µl oligo dT primer with DEPC water in a final volume. Next, we added reaction buffer, dNTP, RNase inhibitor, and reverse transcriptase to the master mix. Reverse transcription was done as follows, incubation for 60 min at 42 C and termination by heating at 70 C for 5 min.

The cDNA was amplified using reverse transcription polymerase chain reaction, and forward and reverse primers of human VEGF-A, MUC1, CSF1, HBEGF, FGF2, and β-actin(Metabion, Martinsried, Germany), and platinum blue PCR super mix (Invitrogen, Paisley, UK). Table I lists the primer sequences. The β-*actin* was used as the housekeeping gene. The PCR protocol was as follows: 5 min at 95 C for initial denaturation, 40 cycles for 45 sec at 95 C, 45 sec at 60 C, and 45 sec at 72 C. Non-template water was used as a negative control in the experiment to ensure that any amplification observed in the PCR products was due to the presence of the target gene and not to contamination or other factors. The PCR products were separated by electrophoresis on a 1.7% agarose gel (Sigma, UK).

### Real-time quantitative PCR (RT-qPCR)

Real-time PCR was used to assess the relative expressions of the study genes in the case and control groups. RT-qPCR to compare the levels of mRNA expression of certain genes in the case and control groups. The synthesized cDNA, Power SYBR Green Master Mix (Applied Biosystems, UK), and the primers are listed in table I. To perform real-time PCR, for each reaction, we added 5 μl SYBR green reagents, 11 μl molecular grade water, 1 μl of each of the forward and reverse primers, and 2 μl cDNA. The amplification conditions, such as the annealing temperature and the number of cycles, were likely optimized including: 10 min at 95 C, 40 cycles at 95 C for 15 sec, and 60 C for 60 sec. The RT-qPCR was performed under standard conditions, which included the use of triplicate samples.

The quantities of relative *VEGF-A*, *MUC1*, *CSF1*, *HBEGF*, and *FGF2* expressions were compared between the case and control groups. Real-time PCR data were analyzed using the relative cycle threshold (CT) method. The ΔΔCT value was calculated by determining the difference between the case and control groups, which allows for the normalization of the data to a reference gene.

**Table 1:** Primers sequences used in the study


**Gene**	**Forward primer**	**Reverse primer**
***CSF1***	5 ' CTGTAGCCACATGATTGGGAGT3 '	5 ' TGTAATTTGGCACGAGGTCTCC3 '
***FGF2***	5 ' CTGTACATTTTTGGGGTCAGCTC3 '	5 ' CCAGCATTTCGGTGTTGAAGAA3 '
***MUC1***	5 ' CAGCCTCTCTTACACAAACCCA3 '	5 ' AGAACCTGAGTGGAGTGGAATG3 '
***HBEGF***	5 ' CATCCCCACAATCTGGCTTAGT3 '	5 ' ACCCCTACATCCTGACCATACA3 '
***VEGF-A***	5 ' GAGAGAAGTCGAGGAAGAGAGAG3 '	5 ' CCCAAAAGCAGGTCACTCACT3 '
*** -actin***	5 ' CAAGATCATTGCTCCTCCTG3 '	5 ' ATCCACATCTGCTGGAAGG3 '
*CSF1*: Colony-stimulating factor-1, *FGF2*: Fibroblast growth factor, *MUC1*: Mucin-1, *HBEGF*:Heparin-binding epidermal growth factor-like growth factor, *VEGF-A*: Vascular endothelial growth factor A		

### Ethical considerations

This study was performed following the Declaration of Helsinki and its latest updates and was approved by the Reproductive Biomedicine Research Center Ethics Committee at Royan Institute, Tehran, Iran (Code: IR.ACECR.ROYAN.REC.1398.072). All participants signed the informed consent forms before participating in the study.

### Statistical analysis

Statistical analysis was performed using SPSS software (version 22; Inc, Chicago, IL, USA). All data were shown as mean ± Standard error (SEM). Normal distribution of the data was evaluated by t2-sample Kolmogorov-Smirnov Test. Data were analyzed using the Mann-Whitney Test for comparing values between case and control groups which were not distributed normally. A p-value < 0.05 was considered statistically significant.

## 3. Results

We examined relative expressions of *VEGF-A, MUC1, FGF2, CSF1, *and* HBEGF *genes in 10 cases (n = 5 per group).

### Real-time PCR assessment of relative gene expressions

**Figure 1:**
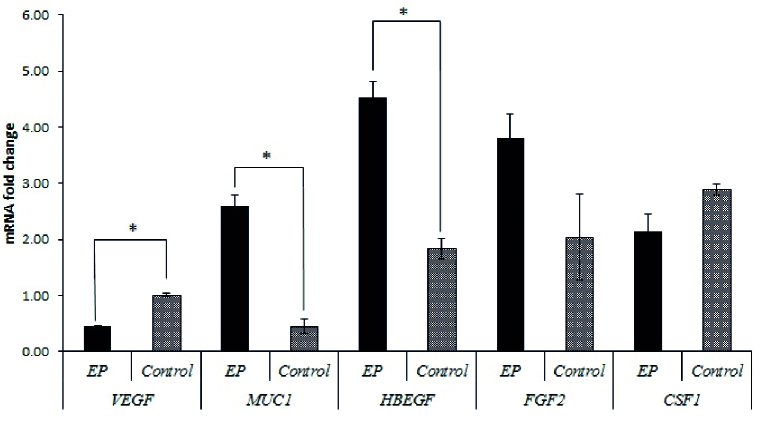
*CSF1, FGF2, HBEGF, MUC1, VEGF-A* gene expression levels in ampulla tissue from EP and controls. Comparison between groups was by analysis of Mean ± SEM. *Bars show the mean and SEM of experiment present, p < 0.05 indicates statistical significance. EP: Ectopic pregnancy,* VEGF-A:* Vascular endothelial growth factor A, *MUC1: *Mucin-1, *CSF1: *Colony-stimulating factor-1, *HBEGF:* Heparin-binding epidermal growth factor-like growth factor, *FGF2: *Fibroblast growth factor 2.

**Figure 2:**
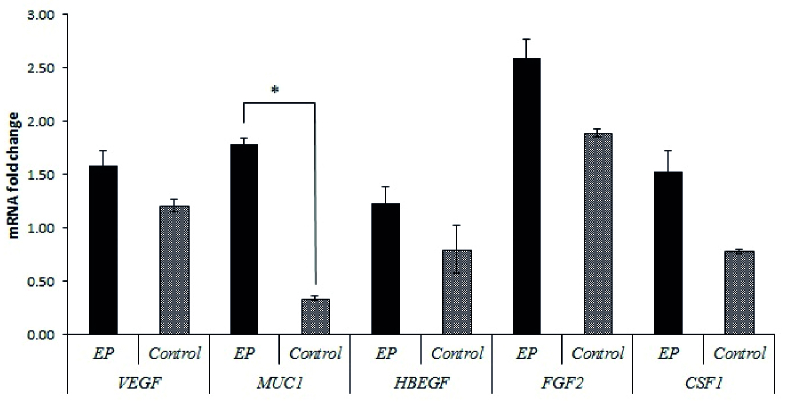
*CSF1, FGF2, HBEGF, MUC1, VEGF-A* gene expression levels in endometrium tissue from EP and controls. Comparison between groups was by analysis of Mean ± SEM. *Bars show the mean and SEM of experiment present, p < 0.05 indicates statistical significance. EP: Ectopic pregnancy,* VEGF-A:* Vascular endothelial growth factor A, *MUC1: *Mucin-1, *CSF1:* Colony-stimulating factor-1, *HBEGF:* Heparin-binding epidermal growth factor-like growth factor, *FGF2: *Fibroblast growth factor 2.

Real-time PCR was used to evaluate the relative gene expressions in the case group compared to the control group. Table II and figures 1 and 2, show the relative expressions of these genes. The results indicated that *VEGF-A *gene expression in the ampulla region was significantly higher in the control group compared to the case group (p < 0.001). No significant difference was observed in endometrial tissue between the 2 groups (p = 0.082). In terms of the *CSF1 *and *FGF2* gene expressions, no significant differences were observed between the 2 groups in both the ampulla and endometrium (p > 0.05). However, expression of *FGF2* in endometrium showed tendency to significant difference (p = 0.057). Furthermore, *HBEGF* gene expression showed a significantly higher expression in the ampulla region of the case group compared to the control group (p < 0.001), but no significant differences existed between these 2 groups in the endometrium (p = 0.193). The only gene that had significantly different expression in both the ampulla and endometrium between 2 groups in both regions was *MUC1,* which indicated higher expressions in the case group for both ampulla and endometrium (p < 0.001).

**Table 2:** The mean expression levels of *CSF1, FGF2, HBEGF, MUC1, VEGF-A* in the endometrium and fallopian tube of case and control


	**Ampulla**			**Endometrium**		
**Gene**	**EP**	**Control**	**P-value**	**EP**	**Control**	**P-value**
	***CSF1***	2.15 ± 0.31	2.89 ± 0.1	0.135	1.53 ± 0.2	0.78 ± 0.02	0.065
***FGF2***	3.82 ± 0.41	2.04 ± 0.76	0.130	2.59 ± 0.18	1.89 ± 0.04	0.057
***HBEGF***	4.53 ± 0.28	1.84 ± 0.18	< 0.001	1.23 ± 0.16	0.80 ± 0.22	0.193
***MUC1***	2.61 ± 0.17	0.46 ± 0.12	< 0.001	1.78 ± 0.06	0.34 ± 0.02	< 0.001
***VEGF-A***	0.45 ± 0.02	1.01 ± 0.03	< 0.001	1.58 ± 0.14	1.21 ± 0.06	0.082
Data presented as Mean ± Standard error (SEM). EP: Ectopic pregnancy, *CSF1: *Colony-stimulating factor-1, *FGF2: *Fibroblast growth factor 2, *HBEGF:* Heparin-binding epidermal growth factor-like growth factor, *MUC1: *Mucin-1, *VEGF-A:* Vascular endothelial growth factor A						

## 4. Discussion

Based on our results, we have assumed that genes involved in implantation including *VEGF-A, MUC1, FGF2, CSF1, *and *HBEGF* might be changed in the endometrium and fallopian tubes during EP.

Our previous investigation showed that *VEGF-A* has higher expression in all regions of the fallopian tubes in pseudopregnant women compared to EP cases. The current study results confirmed this finding. We noted significantly increased *VEGF-A* expression in the fallopian tube of the control group compared with the case group. The decreased expression of *VEGF-A* in EP cases could be due to the effect of hCG on the expressions of *VEGF* and its receptors (2)*. *We could not find any data that evaluated the expression of *VEGF-A* in the endometrial tissue of women with EP. Our data showed no significant difference in *VEGF-A* expression between the 2 groups; however, a slightly higher expression was observed in the EP group in comparison with the control group.

Next, we evaluated the *MUC1 *expression, which had a significantly lower expression in the fallopian tubes of women with EP compared to normal fallopian tubes (19, 20). Another study on EP cases and pseudopregnant women indicated that *MUC1* was localized at the apical surface of the tubal epithelium in both the EP and pseudopregnant groups. These authors also reported significantly lower *MUC1* expression in the EP group (6). A later study confirmed significantly decreased *MUC1* expression in the fallopian tubes of women with EP (14). Interestingly, the results of another study indicated that *MUC1* had higher expression in EP cases (6). These findings could be due to the larger sample size in their study. *MUC1* has an important role in endometrial receptivity. There is decreased *MUC1* expression in women with recurrent implantation failure compared with normal women (14). Our data showed significantly higher *MUC1* expression in both the ampulla and endometria of EP cases compared with controls.

The role of *CSF1* in EP has been investigated. Inflammation is a potential cause of EP, and it was found that macrophages might contribute to the regulation of tubal motility. Decreased expression of *CSF1*, a cytokine that stimulates macrophage production, could cause disruptions in tubal motility, and be involved in EP pathology (15, 21). In the current study, we observed decreased expression of *CFS1* in the ampulla region of the fallopian tubes of EP cases when compared with the controls. In contrast, expression was higher in the endometrial tissue of EP cases compared to controls. However, none of these differences were significant, which might be due to the sample size. Statistical analyses indicated that in larger populations, the difference would become significant.

*HBEGF *is one of the earliest identified molecular mediators of blastocyst implantation and it might play a role in the attachment and penetration steps of embryo implantation (5, 10). We assumed that the expression of this gene could be directly related to a proper implantation process. The results showed a significant increase in *HBEGF* expression in the fallopian tubes of EP cases compared to the controls; however, no difference was observed in the endometria. These findings strongly suggested that the expression pattern of *HBEGF* has a direct impact on implantation.

No significant difference was observed between case and control groups in terms of *FGF2* expression; however, the expressions in both regions from EP cases were higher than the controls. It has been reported that *FGF2* affects endometrium receptivity and proper implantation (8, 9).

## 5. Conclusion

Our results have shown new evidence about the role of *VEGF-A, MUC1, FGF2, CSF1, *and* HBEGF *genes in EP. This is the first study that compares expressions of these genes in both fallopian tube tissue and endometrial tissue. The results strongly suggested that these genes play important roles in proper embryo implantation, and disruptions in their expression patterns could lead to EP. However, more studies should be conducted to confirm the current findings, especially for *CSF1.* Also, an evaluation of the expression patterns in larger populations is recommended to confirm these results.

##  Conflict of Interest

All authors declare that there is no conflict of interest.
